# Insights into the Geomicrobiology of Biovermiculations from Rock Billet Incubation Experiments

**DOI:** 10.3390/life11010059

**Published:** 2021-01-15

**Authors:** Hilary Kelly, Michael N. Spilde, Daniel S. Jones, Penelope J. Boston

**Affiliations:** 1 Earth and Environmental Science, New Mexico Institute of Mining and Technology, Socorro, NM 87801, USA; hilary.kelly@student.nmt.edu; 2 Institute of Meteoritics, University of New Mexico, Albuquerque, NM 87131, USA; mspilde@unm.edu; 3 National Cave and Karst Research Institute, Carlsbad, NM 88220, USA; 4 NASA Ames Research Center, Moffett Field, CA 94035, USA

**Keywords:** biovermiculation, morphological biosignatures, biopattern, cave, corrosion, astrobiology

## Abstract

Biovermiculations are uniquely patterned organic rich sediment formations found on the walls of caves and other subterranean environments. These distinctive worm-like features are the combined result of physical and biological processes. The diverse microbial communities that inhabit biovermiculations may corrode the host rock, form secondary minerals, and produce biofilms that stabilize the sediment matrix, thus altering cave surfaces and contributing to the formation of these wall deposits. In this study, we incubated basalt, limestone, and monzonite rock billets in biovermiculation mixed natural community enrichments for 468–604 days, and used scanning electron microscopy (SEM) to assess surface textures and biofilms that developed over the course of the experiment. We observed alteration of rock billet surfaces associated with biofilms and microbial filaments, particularly etch pits and other corrosion features in olivine and other silicates, calcite dissolution textures, and the formation of secondary minerals including phosphates, clays, and iron oxides. We identified twelve distinct biofilm morphotypes that varied based on rock type and the drying method used in sample preparation. These corrosion features and microbial structures inform potential biological mechanisms for the alteration of cave walls, and provide insight into possible small-scale macroscopically visible biosignatures that could augment the utility of biovermiculations and similarly patterned deposits for astrobiology and life detection applications.

## 1. Introduction

**Figure 1:**
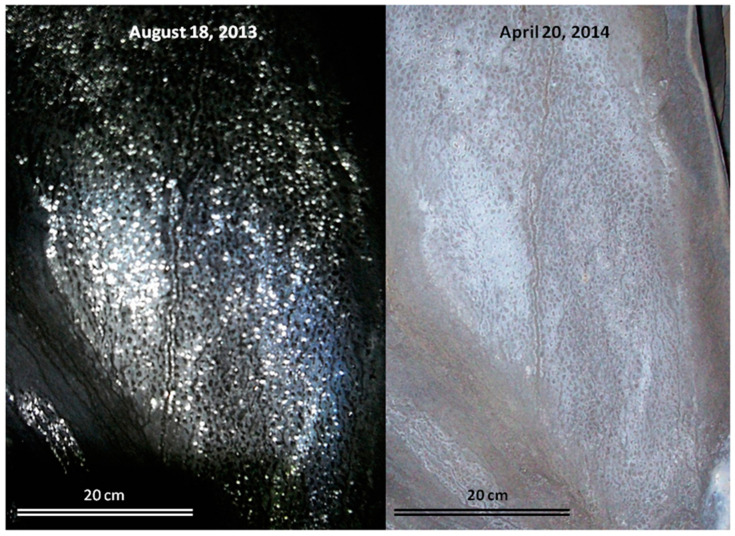
Biovermiculations from Four Windows Cave, El Malpais National Monument, New Mexico, USA. The images were taken from the same location under wet (left) and dry (right) conditions, approximately 8 months apart.

Biovermiculations are geometrically distinct microbial mat communities that occur on cave surfaces [1,2,3,4,5,6]. These formations exhibit a network of “worm-like” tracks and splotches that are typically in positive relief compared to the surrounding substrate (Figure 1). Biovermiculations are known from a variety of cave environments, where they were traditionally referred to as clay vermiculations (e.g., [7]). We use the term “biovermiculation” here *in sensu* Hose et al. [8], who found substantial microbial biomass and biofilm in biovermiculations from sulfide-rich Villa Luz cave. This term is now used more broadly, based on the recognition of a substantial microbial content in vermiculations in many cave systems (e.g., [6,7,9]). Similar formations have also been observed at the sub-millimeter scale in cyanobacterial hypoliths on the underside of a translucent rock in Strzelecki Desert, Australia [10] as well as in higher vegetation patterns in the Negev Desert, Israel that exhibit biovermiculation geometry on a multi-meter scale [11].

Cave biovermiculations are thought to be the result of physical and biological processes. Flocculation of sediments during wetting and drying of damp cave walls and properties of the underlying sediment likely play a role in pattern development [7,12], as do microbial growth characteristics [1,6]. However, the biological factors that contribute to their formation remain significantly less well understood. Insights into the modalities of microorganisms in biovermiculation communities can shed light on why biovermiculations develop where they do and inform cave wall biogeochemistry more broadly.

Biovermiculation microbial communities may alter the mineralogy of the cave wall by corroding the underlying substrate and inducing the formation of secondary minerals. Addesso et al. [7] observed secondary minerals and dissolution features associated with biovermiculations, and there have been multiple reports of microbial biofilm trapping mineral grains within the biovermiculation matrix [5,13]. Some authors have suggested that fungi and other microorganisms in biovermiculations could dissolve carbonate host rock by secreting organic acids [14,15]. Microorganisms in limestone caves can corrode carbonate bedrock by these and other processes including but not limited to carbonic acid production by organoheterotrophic microorganisms, sulfuric acid production by sulfide-oxidizing bacteria and archaea, metal cycling by Fe- and Mn-metabolizing organisms, and microboring by phototrophs [16,17,18,19,20]. Furthermore, biovermiculations occur on a variety of substrates, and to our knowledge, there is little information about how these microbial communities might impact non-carbonate host rock.

Because of their distinctive patterned appearance and wide occurrence on a range of surfaces, biovermiculations are potential morphological biosignatures [21]. Yet characterization of biosignatures based exclusively on morphology is insufficient to distinguish true biosignatures from bioimposters [22]. For morphological biosignatures to be useful for astrobiology and life detection applications, they should be considered in cohort with biosignature “suites” [23] where other evidence in the suite comes from autonomously demonstrable biosignatures based on characteristics other than morphology. Thus, there is a need for an established set of diagnostic criteria to distinguish microbially-produced biomineral features from those of abiotic origins that mimic a similar vermiculated macroscopic pattern [24,25]. Such criteria must include small-scale features like secondary minerals, specific alteration textures in the underlying substrate, and microscopic patterns that are demonstrably biogenic.

Here we report the results of long-term incubation experiments designed to evaluate how biovermiculation microbial communities may alter rock surfaces, in order to identify potential small-scale macroscopically visible biosignatures and test whether microbial alteration could be reproduced in the laboratory for future comparison to cave substrates altered by natural biovermiculations in situ. We suspended rock chips (hereafter referred to as billets) in various dilute growth media that were inoculated with biovermiculation samples for durations ranging from 468 to 604 days, and used SEM to evaluate pre- and post-incubation textural and mineralogical changes. The overarching question driving this work was: how do biovermiculation microbial communities alter rock surfaces, and how might the produced textures represent biosignatures to augment the use of these patterned deposits for life detection efforts? To address this question, we evaluated (1) corrosion of rock billet surfaces, (2) formation of secondary precipitates, and (3) associated biofilm morphologies and other microbial structures. We also compare different drying and preparation methods for SEM imaging, and discuss our results in the context of astrobiological applications of morphological biosignatures.

## 2. Materials and Methods

### 2.1. Rock Billet Collection and Preparation

Rock billets used in the incubations were prepared from basalt, limestone, and monzonite. Basalt samples were collected from Four Windows Cave and Big Skylight Cave, El Malpais National Monument, NM, USA. Limestone samples were collected from Fort Stanton Cave, NM, USA, and monzonite samples were collected from the Capitan Mountains, Lincoln National Forest, NM, USA. Rock samples were cut into billets approximately 2 cm × 5 cm × 1 cm. For identification purposes, the edges of each billet were notched to correspond to its sample number.

### 2.2. Incubation Experiments

Biovermiculations in Four Windows Cave (El Malpais National Monument, NM, USA) were sampled on 15 July 2013. Two vials of each of 6 media types (AIFeMn, R2A + N, RASS, TMC, V3, and V5; see media recipes in Section 2.3, below) were inoculated in the cave using a sterile inoculation loop, and then incubated in a dark fridge at 4 °C to replicate the in situ temperature of Four Windows Cave. Four Windows Cave is a cold, dark environment and temperatures average 2.9–3.5 °C year round at the biovermiculation collection site within the cave. After an incubation period of 16 days, 4 of the 12 biovermiculation enrichments were used to inoculate a batch of 16 tubes containing AI medium—4 replicates for each culture. The remaining 8 enrichments were used to inoculate a second batch of 32 AI tubes in quadruplicate on 21 April 2015. After an incubation period of 32 days and 661 days (“batch 2” and “batch 1,” respectively), the inoculated AI cultures were visually examined and tubes exhibiting the best growth (based on turbidity) were selected to inoculate long-term incubation experiments (Table 1). This strategy was used to ensure initial growth by using diverse media types, and then enrich for actinomycetes prior to long-term incubations, because this bacterial group is especially abundant in wall deposits at Four Windows Cave ([26]).

Long-term incubation experiments were performed in 1 L Fisher Scientific Pyrex™ flasks with sterilized rock samples suspended in 5 types of dilute medium (iron [Fe] carbonate medium, manganese [Mn] carbonate medium, triple sugar iron agar, AI medium, and nutrient broth, described below). These media were selected to encompass a range that included energy sources that might be expected from the host rock (e.g., Fe and Mn carbonates) as well as more general media like nutrient broth that would encourage the growth of diverse populations. Flasks of each medium type contained one billet each of either the basalt (from either Four Windows or Big Skylight), limestone, or monzonite, with the exception of the AI medium flasks, which only contained basalt billets (Table 1). All flasks were inoculated on 23 May 2015, and were harvested after microbial growth became visible in the flasks. Flasks A–F, J, K, and L were incubated for 468 days; Flasks G, H, I, M, N, and O were incubated for 604 days before rock sample harvesting (Table 1). Upon harvesting, subsets of each billet were prepared using each preservation and drying method, as described in the section on SEM methods, below.

Mineral saturation indices for the starting media used for the long-term incubation experiments were calculated using PHREEQC v. 3.3.3 [27] with the Minteq database, based on the inorganic ions added in the salts and assuming dissolved inorganic carbon was initially in equilibrium with the atmosphere. These calculations do not account for metals or other ions that might be present in the agar or other components of pre-made media (e.g., [28]).

### 2.3. Enrichment and Incubation Media

Enrichment experiments used the following 6 media types.

- AIFeMn medium: Iron- and manganese-rich Actinomycete Isolation medium (AIFeMn) was prepared with 22.0 g of Difco^TM^ Actinomycete Isolation Agar powder, 0.5 g FeSO_4_·7H_2_O, 0.3 g of FeCl_3_·6H_2_O, 1.0 g of MnCl_3_·4H_2_O, 0.3 g of MnO_2._, in 1 L deionized water (ddH_2_O), with 5 g glycerol added after autoclaving. This medium is commonly used for the enrichment of iron and sulfur bacteria from soil and water.
- R2A + N medium: R2A + N was prepared by adding add 18.1 g R2A Agar (Oxoid CM 906) to 1 L of ddH_2_O and autoclaved for 20 min at 121 °C. After cooling, 10 mL of Nystatin was added [29]. Target organisms for this R2A + N medium are slow-growing bacteria, thus Nystatin is added to impede fungal growth.
- RASS medium: Reduced Arginine-Starch Salts (RASS) medium was prepared with 1.25 g of starch, 0.1 g of arginine, 1.0 g NaCl, 1.0 g K_2_HPO_4_, 0.5 g of MgSO_4_·7H_2_O, 15.0 g agar, in 1 L of ddH_2_O, with filter-sterilized trace elements (0.001 g CuSO_4_, 0.001 g MnSO_4_, 0.01 g Fe_2_(SO_4_)·6H_2_O, 0.001 g of ZnSO_4_·7H_2_O) added after autoclaving. RASS medium is commonly used for cultivating fungi.
- TMC medium: Trace Metals 44 [30], with carbon source added (0.1% *w/v* acetate). Used for rock-inhabiting organisms that are accustomed to very low organic carbon availability.
- V3 medium: Variant No. 3 (V3) was prepared with 0.5 g KNO_3_, 0.25 g Na_2_HPO_4_·12H_2_O, 0.5 g Ca succinate (3.54 g L^−1^ of succinic acid and 3.3 g L^−1^ of CaCl_2_·2H_2_O), and 15 g Oxoid agar in 1 L ddH_2_O, keeping the medium at pH 7.2. This was then autoclaved for 20 min at 121 °C and cooled to ~55 °C [31]. V3 medium has been used to cultivate bacterial communities from carbonate deposits, and microorganisms grown on this medium often produce visible CaCO_3_ precipitation [32].
- V5 medium: Variant No. 5 (V5) was prepared by mixing 0.25 g Na_2_HPO_4_·12H_2_O, 5.0 g Ca succinate, 2.5 g CaSO_4_, and 15 g Bacto Agar powder (Difco) in 1 L ddH_2_O, keeping the medium at pH 7.2 (modified from [31]). This medium was selected to grow strains or assemblages capable of calcite precipitation [32] but that use a different nitrogen source from the V3 medium.

Long-term incubation experiments used the following 5 media types:

- Fe carbonate medium: Fe carbonate medium was prepared with 0.125 g K_2_HPO_4_, 0.05 g MgSO_4_·H_2_O, 0.0025 g FeCl_3_, 0.025 g FeCO_3_, and 3.75 g Bacto Agar (Difco) in 500 mL of ddH_2_O and adjusted to pH 6.6. This medium was used to provide a source of iron in a form that is commonly found in limestone matrices, as well as to supply an energy source for iron-oxidizing microorganisms.
- Mn carbonate medium: Mn carbonate medium was prepared with 0.125 g K_2_HPO_4_, 0.05 g MgSO_4_·H_2_O, 0.0025 g FeCl_3_, 0.025 g MnCO_3_, and 3.75 g of Bacto Agar (Difco) in 500 mL of ddH_2_O and adjusted to pH 6.6. This medium was used to provide a source of Mn similar to that found in limestone matrices and to provide a substrate for Mn-oxidizing microorganisms.
- TSI Agar: Triple sugar iron agar (TSI Agar) was prepared at half strength by combining 16.25 g of premixed TSI Agar powder (Difco) with 500 mL ddH_2_O in each flask. TSI Agar is customarily used to differentiate gram-negative bacteria of the *Enterobacteriaceae* family based on their fermentation of lactose, sucrose, and glucose and the production of H_2_S.
- AI Agar: Actinomycete Isolation (AI) Agar was prepared at half strength with 5.5 g AI agar powder and 1.25 g glycerol in 500 mL ddH_2_O. AI medium is designed for cultivation of aerobic *Actinomyces* from soil and water.
- Nutrient broth: Nutrient broth was prepared at half strength with 2.0 g premixed Nutrient Broth powder (Difco) in 500 mL of ddH_2_O for each flask. This was used to encourage growth of a wide range of organisms with high carbon and other nutritional requirements.

### 2.4. Scanning Electron Microscopy (SEM)

Prior to incubation experiments, small samples of the prepared rock billets were collected and cleaned by soaking overnight in 95% ethanol. Samples were then vortexed at maximum speed for 20 s, soaked in acetone for 2 h, and vortexed again. Samples were then washed twice again by soaking for 1 h in ddH_2_O and vortexing. Eight samples (two from each selection site: basalt from Big Skylight Cave; basalt from Four Windows Cave; limestone from Fort Stanton Cave; and monzonite from the Capitan Mountains) were then selected for SEM analysis prior to inoculation and incubation experiments (Figure S1). These samples were specifically selected for relative surface flatness and lack of prominent vesicles in the case of the basalt samples.

To establish a baseline surface morphology and roughness profile for these samples prior to the incubation experiment, the samples were examined in a JEOL 5800 LV scanning electron microscope with an accelerating voltage of 15 kV at the University of New Mexico Institute of Meteoritics. Backscattered Electron imaging (BSE) was used to image the samples uncoated in low vacuum mode at 40 Pa of atmospheric pressure. A copper pen was used to place small dots at the end of rasp marks on the surface of the sample as a starting reference point for collecting SEM images. Areas just below the Cu dot (located directly beneath the rasp marks for sample identification) were imaged as a starting reference point in order to compare images to the same areas post-incubation to assess surface alteration. An Oxford ISIS energy dispersive X-ray analyzer (EDX) was used to initially identify the minerals present in the billets.

Following long-term inoculation experiments, rock samples were harvested, cleaned, fixed and dried in order to be imaged with SEM. Rock samples were harvested aseptically. In order to compare preservation techniques and distinguish sample preparation artifacts from biological signatures, billets were prepared for SEM analysis in three ways: by air drying (no fixation), overnight fixation with 2.5% glutaraldehyde, or overnight soaking in simple phosphate buffer (Sigma-Aldrich). Samples that were treated with glutaraldehyde or phosphate buffer were then dried according to one of two methods: Critical Point Drying (CPD) or a Hexamethyldisilazane (HMDS) drying protocol using 98% HMDS [33].

All samples for CPD or HMDS drying were first prepared for SEM in successive 30, 50, 70, 95% ethanol baths in 15 mL centrifuge tubes for five minutes followed by two baths in 100% anhydrous ethanol for 5 min. CPD dehydration was performed by soaking billets in a 1:1 solution of anhydrous ethanol and anhydrous acetone for 10 min, followed by 10 min in anhydrous acetone, and then placed in a plastic sieve column lined with a small piece of lens paper soaked with anhydrous acetone to prevent it from drying during transfer to the Denton Vacuum DCP-1 CPD chamber, where they were dried using the manufacturer’s protocol. HMDS drying was performed by soaking samples in a 1:1 solution of anhydrous ethanol and HMDS for 10 min, followed by 10 min in pure HMDS. Samples were then mounted onto SEM stubs and allowed to dry overnight in a desiccant chamber. Dried samples were sputter coated with 20 nm of gold palladium (Au-Pd) in a EMITECH K950X Turbo Evaporator prior to examination in the SEM.

## 3. Results

### 3.1. Surface Alteration

**Figure 2:**
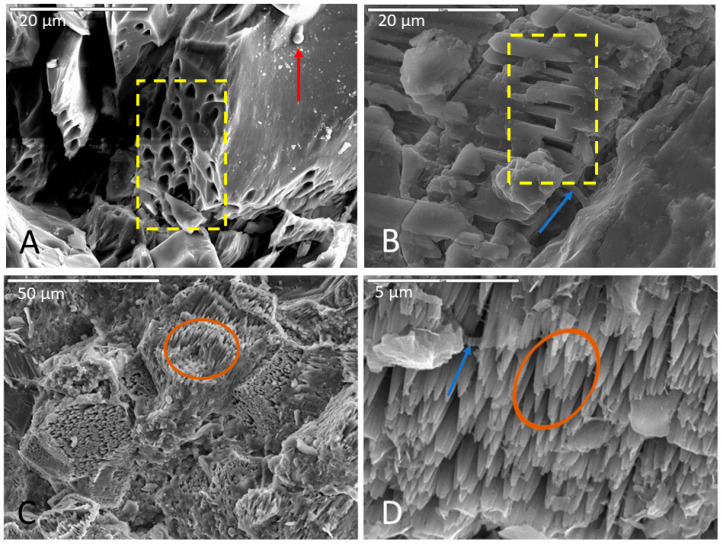
Representative images of surface alteration in (**A**) basalt, (**B**) monzonite, and (**C**,**D**) limestone. (**A**) Corroded olivine crystal in a basalt sample incubated in AI medium (initially enriched in V5 medium), fixed in glutaraldehyde, and dried via HMDS. Note the uniformity and regularity of surface pits (in yellow box), biofilm on the surface of the crystal and inside surface pits, and spherical cell in the upper right of micrograph (indicated with red arrow). Scale is 20 µm. (**B**) Selective corrosion of an Na-feldspar crystal in a monzonite sample incubated in Fe carbonate medium, soaked in phosphate buffer, and dried via HMDS. Note the uniform etch pits within the crystal lattice (inside yellow box) and the closely associated biofilm (indicated by blue arrow). Scale is 20 µm. (**C**,**D**) Highly etched carbonate surface of a limestone sample incubated in Fe carbonate medium, fixed in glutaraldehyde, and dried via CPD. Note the pinnacle towers (inside brown ovals) in both images with biofilm drapes (indicated by blue arrow) in (**D**). (**C**) has a scale of 50 µm and (**D**) has a scale of 5 µm.

Surface alteration was evident in nearly all samples compared to pre-inoculation/incubation SEM images of the same rock samples (e.g., Figure S1). Olivine and sometimes pyroxene crystals in basalts were noticeably more corroded than other minerals, and had a gridded framework, honeycomb-like, or fenestrate appearance, often with a pocked or pitted texture (Figure 2A,B). Pitted surfaces were sometimes associated with biofilm and spherical cells (Figure 2A). Basalt surface alteration showed predominantly as breakdown on the surface with lots of rubble composed of smaller pieces of the same lithology. Many of the basalt samples, however, became noticeably more friable and often broke apart during the harvesting and fixation process.

In monzonite samples, feldspars were consistently more altered than other minerals (Figure 2b). Most samples showed abundant weathering of surfaces and corrosion mostly in Na-feldspars along crystallographic planes (Figure S2). Monzonite samples were also typified by etch pits and surface fractures cross-cutting lamellae, and lamellae were plainly visible (Figures S2 and S3). Corrosion of quartz and albite feldspars were a less common occurrence among monzonite samples. Most often, feldspars were corroded while quartz remained smooth and minimally unaffected (Figures S2 and S3).

Calcite dissolution was consistently observed in limestone samples, often with the formation of highly etched pinnacled surfaces (Figure 2C,D). Surface weathering and corrosion of samples were also common, and limestone samples were somewhat more friable and broke easily at the surface during the post-incubation fixation procedure, although not to the same extent as the basalt billets. Etch pits and dissolution features were also a common occurrence, and in some samples, calcite precipitation occurred as a crust on biofilm surfaces.

### 3.2. Biofilm and Cellular Morphologies

**Figure 3:**
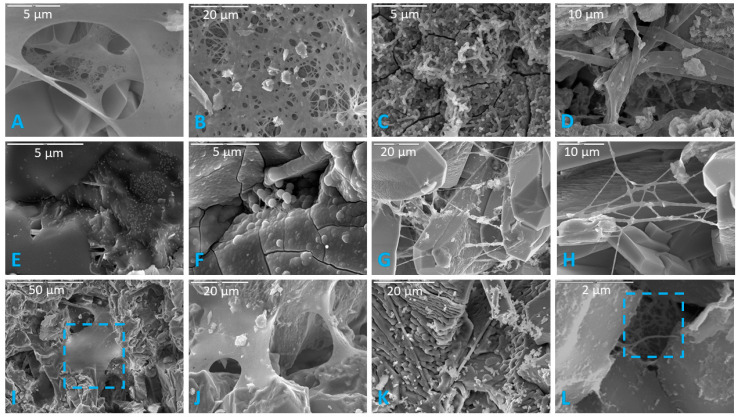
Representative images of the 12 distinct biofilm morphotypes observed. (**A**) Lacy biofilm. Scale is 5 µm. (**B**) Layered biofilm. Scale is 20 µm. (**C**) Filamentous biofilm. Scale is 5 µm. (**D**) Stringy biofilm. Scale is 10 µm. (**E**) Thick biofilm. Scale is 5 µm. (**F**) Thick desiccated biofilm on a basalt sample. Scale is 5 µm. (**G**) Cobweb biofilm. Scale is 20 µm. (**H**) Net-like biofilm. Highly branching net-like biofilm with clay sheets in a limestone sample. Scale is 10 µm. (**I**) Continuous swath (unperforated) biofilm. Blue square indicates continuous/unperforated section of biofilm. Scale is 50 µm. (**J**) Smooth with aggregates (or precipitated minerals). Scale is 20 µm. (**K**) Brain-like (or folded) biofilm. Scale is 20 µm. (**L**) Septate biofilm in a basalt sample. Blue square indicates septate portion. Scale is 2 µm.

Microbial colonization of rock surfaces during the incubation period was evident from the development of biofilms, often in association with filaments and cells on surfaces of samples. Biofilms were observed in majority of samples, regardless of lithology, incubation medium, or inoculum (Table 2). These biofilms varied greatly in abundance and appearance, from robust thick forms, to more delicate net- and cobweb-like forms, to filamentous and even “brain like” in one case (Figure 3). Some biofilms contained many spherical cells while others contained secondary minerals. Based on morphology observed under SEM, we classified the biofilms into twelve distinct morphotypes: lacy, layered, filamentous, stringy, thick, thick and desiccated, cobweb, net like (which is distinguished from the cobweb morphotype by thicker interconnecting strands (Figure 3G,H), continuous swath (unperforated), smooth with aggregates, brain like, and septate. Figure 3 shows a representative example of each morphotype. The “lacy” and “layered” morphologies may well be preparation artifacts attributable to the dehydration effects of the procedures used, while the “stringy” type refers to true filamentous structure which is also often associated with pleomorphic strains [34].

Fixing and drying protocols exerted subtle effects on the abundance of preserved biofilms. A general trend was noted that biofilms were more abundant in HMDS-dried samples, but often had a desiccated appearance. Thick biofilms in samples dried via the HMDS method exhibited pseudo-hexagonal desiccation cracks similar to mud cracks. The HMDS drying method also seemed to better preserve biofilm where an abundance of cells were co-located. The CPD drying method proved better at preserving thicker biofilms while air-dried samples seemed to best preserve individual cells (i.e., not incorporated into a biofilm).

**Figure 4:**
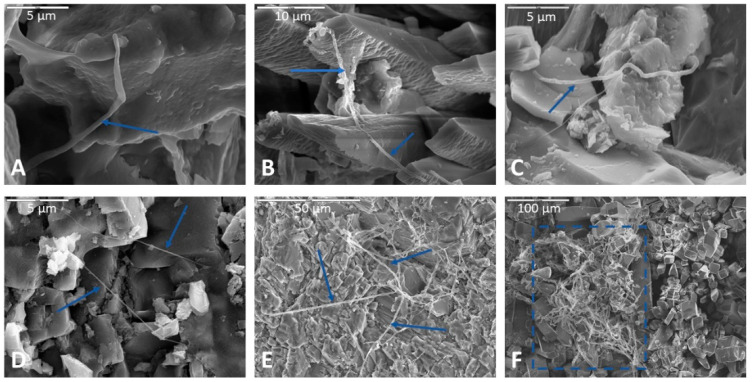
Representative images of filaments observed following incubation experiments. (**A**) Filament at magnification 10,000× in an air-dried limestone sample incubated in Fe carbonate medium. Biofilm spans two rock surfaces in upper left. Scale bar is 5 µm. (**B**) Filaments draped across apatite crystals in another limestone sample incubated in Fe carbonate medium, soaked in phosphate buffer, and dried via HMDS. Scale bar in upper left corner is 10 µm. (**C**) Filament in a basalt sample incubated in AI medium, fixed in glutaraldehyde, and dried via HMDS. Scale bar (upper left of image) is 5 µm. (**D**) Two filaments interwoven and incorporated into two separate swaths of biofilm (lower right of image and upper left of image, indicated by blue arrows) on the surface of a basalt sample. A continuous smooth swath of biofilm can be seen each in the upper left and lower right portions of the micrograph. This basalt sample was incubated in AI medium, fixed with glutaraldehyde, and dried via HMDS before processing for SEM. Scale bar in the upper left of the image is 5 µm. (**E**) Filamentous web on the feldspathic surface of a monzonite sample incubated in nutrient broth, soaked in phosphate buffer, and dried via HMDS. Scale is 50 µm. (**F**) Filamentous mass strung across apatite crystals (indicated by blue box) on the surface of limestone incubated nutrient broth and soaked in phosphate buffer and dried via HMDS. Scale is 100 µm.

Filaments were present in approximately 60% of samples, based on SEM observations. Rock billets incubated in AI medium produced the most filaments. The order of decreasing filament abundance was as follows: AI agar > nutrient broth > Fe carbonate medium > Mn carbonate medium > TSI agar. The fewest number of filaments were observed on rock billets incubated in TSI agar. Samples soaked in phosphate buffer prior to drying showed greater abundance of filaments compared to those fixed in glutaraldehyde. The air-drying method preserved filaments least well of the three drying methods. Figure 4 shows some representative examples of the filaments that were observed.

**Figure 5:**
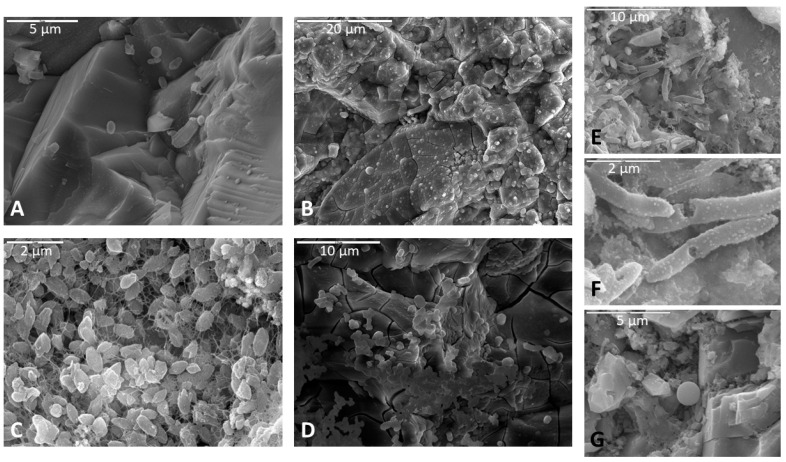
Representative images of cells observed on rock billets following incubation. (**A**) Spherical cells in a monzonite sample. Scale is 5 µm. (**B**) Many spherical cells incorporated into a thick biofilm in a basalt sample. Scale is 20 µm. (**C**) Bacilli in lacy biofilm on a basalt sample. Scale is 2 µm. (**D**) Some spherical cells and bacilli over a thick desiccated biofilm in a basalt sample. Scale is 10 µm. (**E**) Bacilli associated with biofilm on a basalt sample. Scale is 10 µm. (**F**) A closer image of image (**E**) showing some surface details of bacilli. Scale is 2 µm. (**G**) Spherical cell closely associated with a lacy biofilm on a basalt sample. EDS data confirmed a high carbon peak (not shown). Scale is 5 µm.

Diverse cellular morphologies were observed, including cocci, bacilli (i.e., rod-shaped), coccobacilli (rounded rods), diplococci, streptobacilli like, streptococci like, and filamentous. Unsurprisingly, samples incubated in nutrient broth exhibited the most diversity in cellular morphology. The abundance and morphology of observed cells varied with each rock sample type (Table 1, Figure 5). The drying method had a substantial impact on cell preservation, with very few or no biofilm-associated cells preserved with air drying. However, more isolated cells were observed using this method.

### 3.3. Secondary Minerals

**Figure 6:**
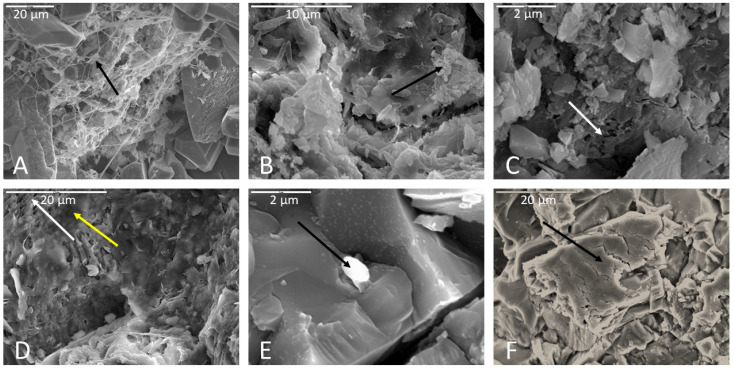
Representative images of secondary minerals associated with rock surfaces. (**A**) Arrow indicates clays associated with a biofilm on a limestone sample. Scale is 20 µm. (**B**) Iron oxides in a biofilm on a basalt surface (detail in Figure S4). Scale is 10 µm. (**C**) A calcium-rich crust precipitated on a limestone sample incubated in Fe carbonate medium. Scale is 2 µm. (**D**) Yellow arrow indicates illite clay on the weathered surface of limestone. White arrow points to an area of calcite dissolution. Scale is 20 µm. (**E**) Ti-oxide attached to biofilm covering sodium plagioclase on a basalt sample. Scale is 2 µm. (**F**) Silica crust on a basalt sample. Scale is 20 µm.

Secondary minerals produced during the experiment included clays, iron oxides, calcium and silica crusts, and apatite, based on crystal form and elemental abundance in EDS. Clays were observed that were associated with limestone surfaces, often within biofilms (Figure 6A). Illite was present in association with partially dissolved calcite (Figure 6D). Calcite crusts (Figure 6C) were often observed in depressions of areas of significant limestone corrosion. Iron oxides were commonly associated with biofilms on basalt samples (Figure 6B). Silica crusts formed on some basalt samples post-inoculation/incubation (Figure 6F). Biofilms appeared to be less prevalent wherever silica crusts were present.

**Figure 7:**
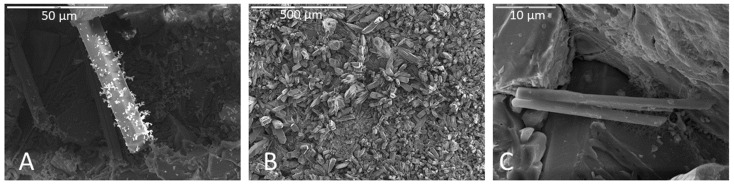
(**A**) Apatite crystal covered in phosphatic residue on a basalt sample incubated in Mn carbonate medium and soaked in phosphate buffer. Scale is 50 µm. (**B**) Surface of a limestone sample incubated in Fe carbonate medium and soaked in phosphate buffer with abundant apatite crystals. Scale is 500 µm. EDS provided in Figure S5. (**C**) Apatite crystal growth on a monzonite sample incubated in Fe carbonate medium and fixed in glutaraldehyde instead of soaking in phosphate buffer. Apatite crystals are attached to biofilm and in close proximity to cells. Scale is 10 µm.

Apatite was the most common secondary mineral observed. Ubiquitous acicular clusters of calcium phosphate crystals were observed on limestone samples post-inoculation and incubation that were not present prior to inoculation and incubation (Figure 7B). These features are possibly the result of precipitative effects of the sample soaking agent (phosphate buffer) (Figure 7A); however, direct evidence of precipitated phosphatic minerals within biofilm was also observed in many samples not soaked in phosphate buffer (e.g., Figure 7C).

## 4. Discussion

### 4.1. Implications for Biological Substrate Alteration

Microorganisms can play a role in cave formation and subsequent enlargement by dissolving limestone and other rock surfaces [35,36,37]. Biovermiculations are widely known from limestone caves, and are also found in lava caves and cavities in other lithologies. In these settings, the microorganisms that inhabit biovermiculations could corrode the underlying substrate indirectly by producing acids as a byproduct of metabolic activities, or directly by actively mobilizing metals or other compounds in the rock matrix. In the case of lava caves, the basalt host rock contains abundant mineral substrates with reduced iron (e.g., olivine, pyroxene) that has been shown to provide an energy source for chemolithotrophic microorganisms [38,39]. Microbial “mining” for reduced iron and manganese in the carbonate matrix has also been implicated in wall corrosion in limestone caves [20,40] and in other iron-manganese rich minerals (e.g., [41]). Further, biological iron reduction has been proposed to play a role in the formation of iron ore caves [42]. Feldspar has been shown to be significantly altered by heterotrophic organisms [43], a wide variety of bacteria (e.g., [44]), and lichens [45]. The surface alterations that were observed in this study can offer insight for potential mechanisms of biocorrosion associated with biovermiculation microorganisms.

**Figure 8:**
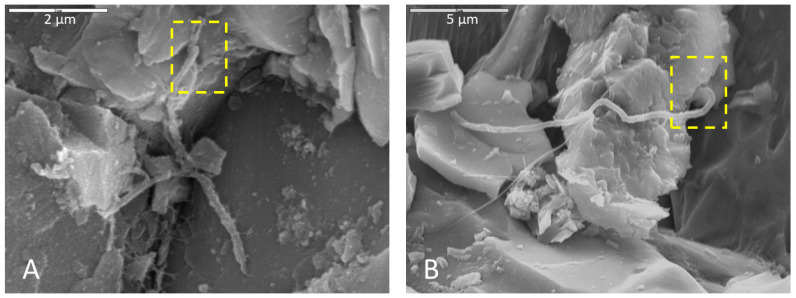
Microbial filaments in basalt samples incubated in AI medium (initially enriched AlFeMn (**A**) and V5 media (**B**)), soaked with phosphate buffer and glutaraldehyde for (**A**) and (**B**), respectively, and dried with HMDS. Yellow boxes show terminus positions of filaments where they appear to be boring into natural crevices in the rock surfaces, possibly expanding existing fractures. (**A**) Scale 2 µm. (**B**) Scale is 5 µm.

Olivine within basalt billets was consistently corroded in incubations using all media types. Pitted surfaces were sometimes associated with biofilm and spherical cells, indicating that some of this corrosion could be biological. For example, the corroded olivine crystal in Figure 2A exhibits uniform regularity of semi-circular polygonal pockets that could be indicative of bacteria accessing iron in the crystal structure. As another example, Figure 8 shows two separate samples of basalt, both collected from the same cave and both incubated in AI medium, where filaments appear to be attached at natural surface depressions or crevices. The surfaces of basalt billets could have been altered by filaments boring into natural crevices and expanding existing fractures, increasing friability.

Olivine is unstable under Earth surface conditions, and is one of the first minerals to weather [46]. In some basalt samples, olivine dissolution was accompanied by dissolution of pyroxenes as well as that of other heavy metal oxides. In Supplementary Figure S6, an arrow indicates a decrepitated Fe–Ti oxide in a basalt sample in which dissolution of Mg-rich pyroxene and olivine occurred locally along crystallographic planes. The order of weathering in basalt without glass is olivine, labradorite, augite, and finally Fe–Ti oxides [47], so this altered Fe–Ti oxide potentially implies that multiple minerals in this basalt sample were highly corroded. This same sample also contained a biofilm with a clear Mg peak immediately adjacent to a corroded pyroxene with no Mg (Figure S6); other pyroxene crystals in the sample with no associated biofilm had clear Mg peaks (Figure S6), potentially indicating that Mg was selectively removed from the mineral and accumulated in the biofilm.

In incubations with monzonite billets, feldspars were typically more altered than other minerals (Figure 2B), and sodium feldspars exhibited the most corrosion along crystallographic planes. Filaments, cells, and biofilms in fractures and corrosion pits where feldspars were altered could potentially indicate microbial utilization of nutrients such as potassium, sodium, or calcium. Biofilms were observed settled in depressions and crevices. It is possible that biofilms could enhance corrosion at crystal margins where these natural depressions and crevices in the rock surface commonly occur. However, feldspars typically weather at a faster rate compared to other minerals in the matrix like quartz, and networks of filaments were observed on unaltered feldspars as well, thus any claim of biogenicity requires further confirmation.

In incubations with limestone billets, the surfaces exhibited dissolution in the form of highly etched, pinnacle-like textures. In some samples, the tips of the pinnacles had etch pits with biofilms incorporated in the fabric. Etch pits were not observed in areas without biofilm drapes, often on the tips of the pinnacles, so biofilm formation possibly sped up the etching process. Clays were observed in some samples, often located within the biofilm or on the tips of the pinnacles in an etched carbonate surface. The source of the silicates for clay formation is presumably impurities in the limestone.

In addition to corrosion, biovermiculation microorganisms could also alter their substrate by inducing the formation of secondary minerals. Microorganisms have been implicated in the formation in certain speleothems and other cave-hosted mineral deposits, by directly or indirectly precipitating carbonates [48,49,50], sulfates [51], silica [52], iron- and manganese oxides [53], and other minerals [19,37]. In some cases, we observed associations between secondary minerals and microbial structures that could indicate a biological role in their formation. For example, we identified iron-rich secondary minerals incorporated into the upper portion of a biofilm in a basalt sample incubated in Fe carbonate medium and soaked in phosphate buffer (Figure 6B), associated with coccobacilli directly beneath the iron precipitates. In another example, Figure 6E shows ilmenite attached to a biofilm on a Na-plagioclase host crystal in a basalt billet. Fe-oxidizing or Fe-scavenging bacteria could have contributed to these Fe(III) precipitates in the biofilm (e.g., [54]). We also observed cauliflower-like carbonate precipitates that were depleted in Mg relative to the uncorroded carbonate (Figure S7), indicating selective corrosion and/or reprecipitation.

Some of the corrosion textures or secondary mineral precipitates we observed are also likely abiotic artifacts. Saturation indices calculated for the incubation media (Table S1) show that media likely started out supersaturated for many iron oxides and iron phosphates, and undersaturated for manganese oxides and sulfates. Interestingly, although we observed limestone corrosion in all media types, the Fe and Mn carbonate media was initially undersaturated for carbonate phases while TSI and AI were supersaturated. This suggests that while iron oxide formation may not have been directly microbial, limestone corrosion may have been. However, we caution that, because abiotic controls were not performed, we cannot say which of the textures and minerals described above are biologically influenced. Nevertheless, the specific associations between many surface textures and microbial biofilms, cells, and filaments that we observed suggests some amount of direct or indirect biological mediation. These corrosion features and secondary minerals produced in the laboratory will provide a valuable starting point for future studies aimed at identifying alteration textures on cave surfaces associated with biovermiculation microbial communities in situ.

Biovermiculation microbial communities have been studied using rRNA gene sequencing from multiple limestone caves, and typically contain diverse microbial communities dominated by *Proteobacteria*, *Actinobacteria*, *Acidobacteria*, and several other phyla [5,9,55,56,57]. However, less is known about biovermiculations from lava caves. Although biovermiculations from Four Windows Cave have not yet been studied using culture-independent techniques, other communities from this cave have abundant *Actinobacteria* as well as *Proteobacteria*, *Acidobacteria*, and *Nitrospirae* that represent chemoorganotrophs as well as some chemolithotrophic populations that are likely involved in cycling N, Fe, and other elements [26,58,59], consistent with studies of lava cave communities worldwide (e.g., [60,61] and references therein; see [62] for a review).

### 4.2. Preservation Methods

Following incubation, rock billet samples were dried according to one of three methods: air dry, CPD, or HMDS. Samples that were dried by CPD or HMDS were first prepared for SEM by fixation with either glutaraldehyde or phosphate buffer. Fixing agents may have had some effects on rock billet surface alterations and precipitation or formation of secondary minerals, such as the formation of apatite crystals in samples treated with phosphate buffer (Figure 7A,B). However, samples fixed in glutaraldehyde also produced apatite crystals (Figure 7C) but without any of the phosphatic residue observed on the surface of preserved biofilms in phosphate buffer-soaked samples.

In air-dried samples, thin biofilms were common, and appeared to be preserved such that finer details of biofilm morphology could be observed. Filaments and thick biofilms were not well preserved in air-dried samples compared to the CPD and HMDS drying methods. Coccoidal cells not associated with biofilm were best preserved in air-dried samples, and often had a dimpled or flattened appearance, presumably due to desiccation.

In CPD-dried samples, thick biofilms were well preserved with minimal desiccation compared to HMDS-dried samples in which thick biofilms more often displayed pseudo-hexagonal desiccation cracks similar in appearance to mud cracks. Because of the preservation potential of thicker biofilms in CPD samples, more morphotypes of biofilms were observed. Thin biofilms were well preserved, particularly in lacy biofilms where they were netting together coccobacilli cells to an underlying thicker biofilm. More coccobacilli were observed in CPD samples compared to other drying methods.

Biofilms were more abundant in HMDS samples compared to other drying methods but fewer morphotypes were observed. Thick (Figure 3E) and thick desiccated (Figure 3F) were the most common biofilm morphotypes observed in HMDS. HMDS appears to have a highly desiccating effect on biofilms, which would explain why thick biofilms were more common, and usually had desiccation cracks on their surfaces. Thick biofilms nearly always had coccoidal cells incorporated into the biofilm matrix. These biofilm-incorporated cells were best preserved in HMDS samples, but isolated coccoidal cells were best preserved in air-dried samples. Filaments appeared to have been better preserved in HMDS samples compared to other drying methods. Samples soaked in phosphate buffer seemed to have more filaments than those fixed in glutaraldehyde, indicating a preservational artifact associated with the fixation method. Based on these observations, CPD appears to be the best method for preserving diverse biofilm morphologies, while HMDS may be better for quantifying biofilm and filament abundance. Fratesi et al. [63] found that different dehydration and drying techniques produced significant differences in textures and distributions of organic material in prepared SEM samples and concluded that alcohol dehydration and air drying after HMDS infiltration best preserved mucilaginous material. Critical point drying preserved bacteria but stripped away mucilaginous material, which, however, revealed otherwise invisible filamentous structures within the biofilm [63].

### 4.3. Implications for Morphological Biosignatures and Life Detection

**Figure 9:**
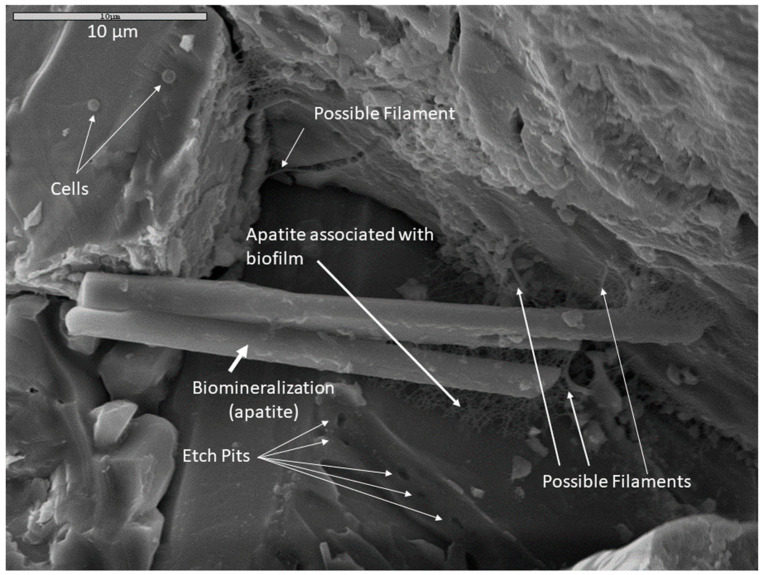
An example of a possible microscopic bioassemblage with multiple features indicating microbial activity. This sample was incubated in Fe carbonate medium, fixed in glutaraldehyde and dried by HMDS. The composition of the large apatite crystal was confirmed with EDS. Scale is 10 µm.

Multiple lines of evidence indicating microbial activity on altered rock surfaces suggest that the observed surface alterations in this study were, at least in part, biologically mediated. Figure 9 is an example of multiple co-occurring indicators of biologic activity in a monzonite sample incubated in Fe carbonate medium and fixed in glutaraldehyde. Cells, possible filaments, and biofilm are present on mineral surfaces. Secondary apatite crystals are present in biofilm (note that phosphate buffer was not used as a soaking agent for this sample, so the apatite was likely precipitated during the incubation period). In a crystal face beneath the apatite crystals, systematic parallel etch pits were observed which could indicate microbially-induced corrosion, perhaps “mining” for nutrients. Taken together, these co-located mineralogical, textural, organic, and cellular indicators of microbial activity could represent a microscopic biosignature suite.

Distinctly-patterned macroscopic morphological features that can demonstrably indicate past or extant microbial life, and are able to be preserved within the rock record, would be immeasurably valuable for life detection on other planets. Biovermiculations are one example of a biopattern which can be defined as an easily recognizable and geometrically-distinct pattern or network of patterns on surface materials that are biologically derived or influenced [23,24,25,64]. These can occur in multiple discrete environments at many different spatial scales. Biopatterns are one broad category of biosignature [21,24,25,65,66,67], specifically a subset of morphological biosignatures [25,64] that when accompanied by other biological indicators may comprise unified biosignature assemblages. For life detection missions, biopatterns could be considered detectable micro or macro-patterns that evince evidence of microbial presence or previous processes [64]. As an example, in the case of chenille spar (a known biothem, i.e., a mineral cave deposit that has been influenced by life), palisades fabric (Figure S8) is proposed as a microbially-produced geometric pattern type of a micro and macro-biopattern detectable by pattern recognition software [64,68,69]. In the limestone samples incubated in this study, parallel spiked protrusions, digitate features, and other small-scale palisades fabrics (not observed before inoculation and incubation) were the most dramatic surface alteration features seen (Figure 2C,D). However, more SEM work on known non-microbially-altered limestone is needed to establish relevancy and to ascertain if similar prismatic carbonate crystals and nm–µm scale palisades fabrics could also be generated by abiotic dissolution.

Physical/chemical alteration of mineral and rock surfaces facilitated by microorganisms has relevance for life detection missions on other planets where any signs of life are likely to be microbial [70] and traces of former life are of great interest (e.g., [71]). If we can detect small and large-scale biopatterns at lower cost with robotic first-line reconnaissance missions, biopatterns—these hopefully unique or at least characteristic morphological biosignatures—should indicate areas suitable for deeper investigation to search for bioassemblages and other biomarkers indicating extinct or extant microbial life. Attention must be paid to the potential for false positives to distinguish potential biotic from abiotic processes (e.g., [22,72]). Thus, future work must rigorously continue to attempt to distinguish between patterns produced with the mediation of biological processes versus any similar patterns that can be demonstrably attributed to abiotic processes. In this study, we observed microscopic bioassemblages that indicate microbial activity and could be used to validate biological processes involved in larger-scale features in future work. The new observations reported here of diverse biofilm morphologies, mineral corrosion textures, and secondary mineral production represent additional ways in which microorganisms could enhance biovermiculation or other biopattern formation, and are features that could represent targets for future in situ investigations both on Earth and life detection missions to other Solar System bodies.

## Figures and Tables

**Table 1:** Summary of long-term incubation experiments.

Flask Identifier	Medium	Rock Type ^1^	Inoculum	Incubation Period
A	Fe Carbonate	Limestone	RASS	468 days
B	Fe Carbonate	Monzonite	V5	468 days
C	Fe Carbonate	Basalt (BS)	TMC	468 days
D	AI	Basalt (4W)	AIFeMn	468 days
E	AI	Basalt (4W)	RASS	468 days
F	AI	Basalt (BS)	V5	468 days
G	Mn Carbonate	Basalt (4W)	R2A + N	604 days
H	Mn Carbonate	Monzonite	V5	604 days
I	Mn Carbonate	Limestone	RASS	604 days
J	Nutrient Both	Monzonite	R2A + N	468 days
K	Nutrient Both	Limestone	V5	468 days
L	Nutrient Both	Basalt (4W)	RASS	468 days
M	Triple Sugar Fe Agar	Basalt (4W)	V5	604 days
N	Triple Sugar Fe Agar	Monzonite	RASS	604 days
O	Triple Sugar Fe Agar	Limestone	AIFeMn	604 days

^1^ BS = Big Skylight Cave; 4W = Four Windows Cave.

**Table 2:** Summary of SEM observations.

Media Type	Fe Carbonate			AI Media	Mn Carbonate			Nutrient Broth			TSI Agar		
Rock Type	Basalt	Limestone	Monzonite	Basalt	Basalt	Limestone	Monzonite	Basalt	Limestone	Monzonite	Basalt	Limestone	Monzonite
Surface corrosion	Abundant	Prevalent	Abundant	Abundant	Abundant	Prevalent	Prevalent	Prevalent	Prevalent	Prevalent	Abundant	Abundant	Abundant
Etch pits observed?	none noted	yes	yes	none noted	yes	none noted	yes	yes	none noted	yes	yes	yes	yes
Specific minerals or other corrosion features	corroded olivine surfaces	corroded calcite (weathered pinnacles, etch pits)	corroded Na-feldspar, sometimes associated with biofilm.	corroded olivine and some feldspar, pyroxene, and Fe-Ti oxides	corroded feldspars	corroded calcite (weathered pinnacles)	parallel etch pits	conchoidal fracturing; isolated etch pits with uniform geometry and size	corroded calcite	corrosion of quartz and feldspars. Feldspar corrosion sometimes associated w/ filaments	surface breakdown, surface etching and surface pits	corroded calcite (pinnacle weathering, sometimes associated w/ etch pits and biofilms)	corroded feldspars
Secondary minerals ^1^	silica crust (containing Si, Ca, Mg, and O); Fe-rich precipitates	illite	apatite crystals, associated with biofilm	apatite crystals (Glut_HMDS), sometimes fibrous or straw-like; iron oxides	none noted	none noted	none noted	ilmenite associated with biofilm; apatite (in Glut_CPD)	apatite (Glut_HMD); Possible calcite (Glut_HMDS); clays	clays; possible carbon-enriched albite	none noted	none noted	none noted
Biofilm Abundance	Common	Moderate	Moderate	Moderate	Minimal in air-dried sample; moderate in Glut_CPD and Phos_CPD	Minimal in air-dried; moderate in Glut_CPD; common in Phos_CPD	Abundant	Moderate	Common; thick biofilm covered most surfaces.	Moderate	Moderate	Minimal, only in air-dried	Abundant
Biofilm Morphotypes ^2^	A, B, C, D, E, F, G, I, J, L	A, B, D, E, G	A, C, D, E, I, J	A, B, C, D, E, F, G, H, I, L	A, B, E, F, G, H, I, J, K, L	B, D, E, I	A, B, D, E, G, H, I	D, E	A, B, C, D, E, G, H	A, D, E	A, B, C, E, H, I, J, L	E	A, B, D, E, G, H, I, J, L
Cell Types Present	cocci and bacilli	coccobacilli and cocci	cocci, diplococci, streptococci, coccobacilli and bacilli	cocci w/ dimpled appearance; coccobacilli; bacilli	cocci; coccobacilli (very prolific in some); some bacilli and streptococci	flattened cocci and coocobacilli (none in air dried sample)	coccobacilli most abundant; some bacilli and cocci	cocci, coocbacilli, bacilla; very abundant in Phos_HMDS sample	cocci; none noted in Phos_CPD sample	cocci, coccobacilli, bacilli	cocci	cocci	cocci, coccobacilli
Filaments Present	Yes, except in air-dried	Yes	Yes	Yes	Yes	Yes, except in air-dried	none noted	Yes, only in air dried	Yes, only in Phos_HMDS	Yes	Yes	Yes, only in air dried	Yes, only in Phos_CPD

^1^ The presence of apatites are only recorded here from samples that did not use phosphate buffer. ^2^ Biofilm morphotypes refer to the following: A-Lacey, B-Layered, C-Filamentous, D-Stringy, E-Thick, F-Thick/Desiccated, G-Cobweb, H-Net-like, I-Continuous swath (unperforated), J-Smooth w/ aggregates, K-Brain-like, L-Septate.

## Supplementary Materials

The following are available online at https://www.mdpi.com/2075-1729/11/1/59/s1, Figure S1: SEM images prior to inoculation, Figure S2: Details of monzonite billets, Figure S3: Details of montonite billets, Figure S4: Details of panel B in Figure 6, Figure S5: Details of panel B in Figure 7, Figure S6: Basalt weathering features, Figure S7: Mg depletion in a corroded limestone, Figure S8: palisades fabric in a corroded monzonite sample, and Table S1: Saturation indices.
