# Supplementary material for: Insights into the Geomicrobiology of Biovermiculations from Rock Billet Incubation Experiments
Source: Life (Basel). 2021 Jan 15;11(1):59. doi: 10.3390/life11010059 (PMC7830032; doi:10.3390/life11010059)
Supplement: Supplementary file 1 [file life-11-00059-s001.pdf]

life

MDPI

Supplementary Materials

Insights into the Geomicrobiology of Biovermiculations from Rock Billet Incubation Experiments

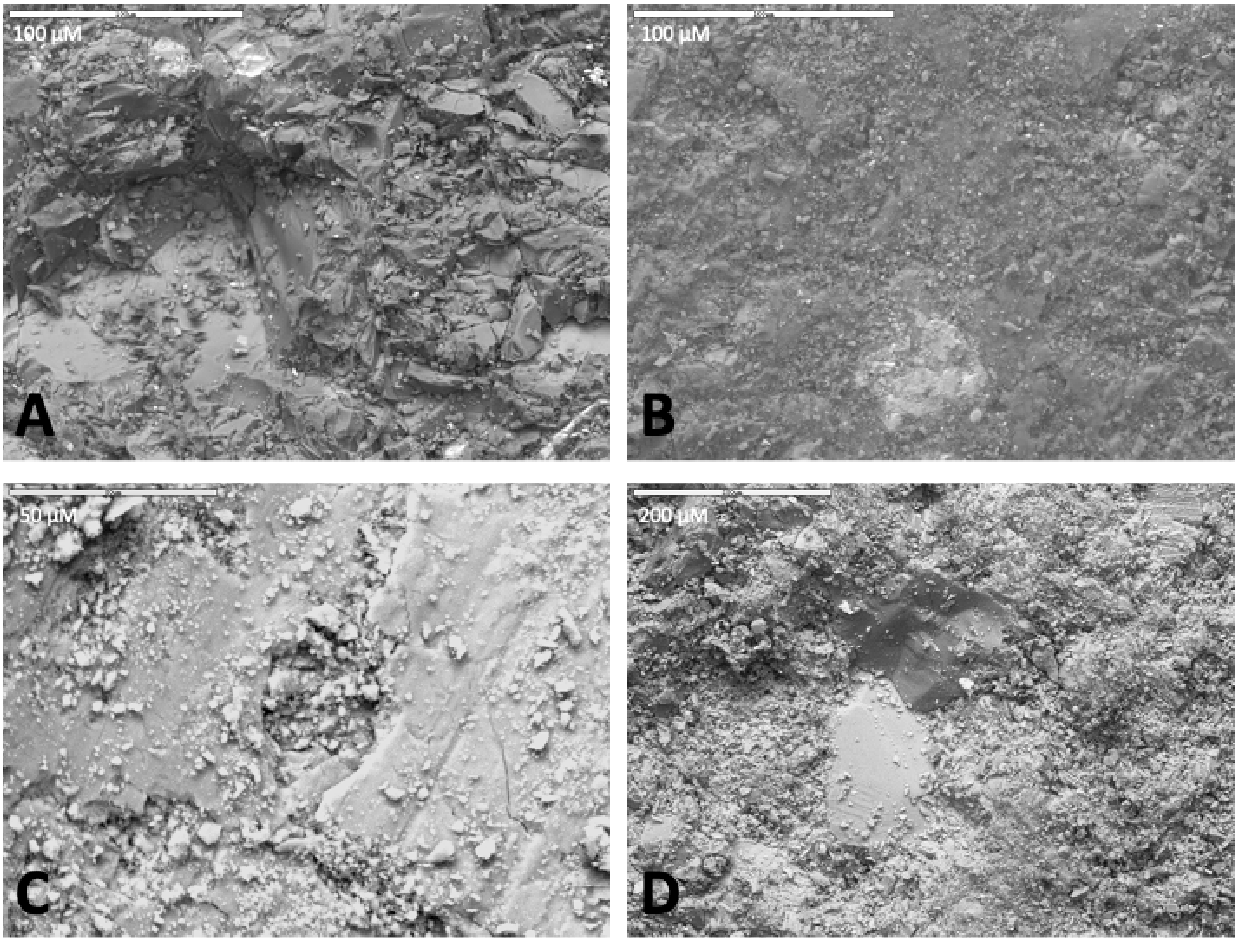

Figure S1. Representative SEM images of rock billet surfaces prior to inoculation. (A) Basalt from Four Windows Cave, scale bar is 100 µm. (B) Basalt from Big Skylight Cave, scale bar is 100 µm. (C) Limestone, scale bar is 50 µm. (D) Monzonite, scale bar is 200 µm.

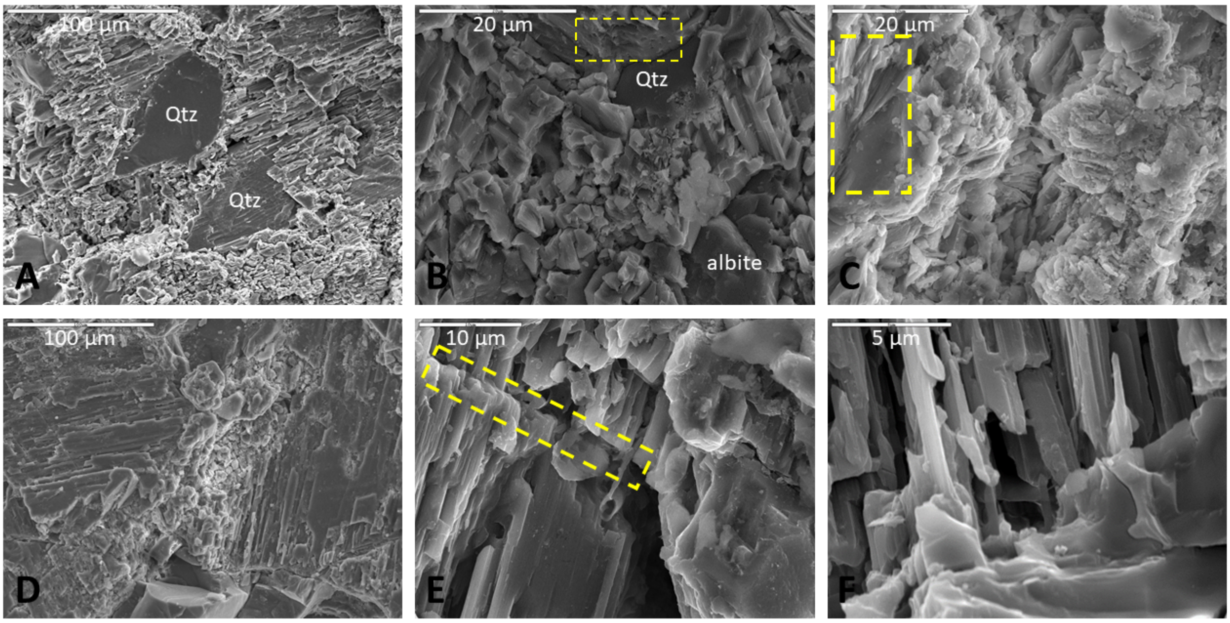

Figure S2. SEM photomicrographs showing alteration of feldspars versus other minerals (panels A and B). Most samples showed abundant weathering of surfaces and corrosion mostly in Na–feldspars along crystallographic planes (C; inside yellow box) and at crystal margins (D). Monzonite samples were also typified by etch pits (B; inside yellow box) and surface fractures cross–cutting lamellae (E; indicated inside yellow box). Lamellae were plainly visible (F). Corrosion of quartz and albite feldspars were a less common occurrence among monzonite samples (B). Most often, feldspars were corroded while quartz remained smooth (A and B). Images A–E are from billets incubated in Fe carbonate medium, F is from a billet incubated in nutrient broth. Images from panels A, B, and F were air dried; panels C, D, and E were soaked in phosphate buffer and dried with HMDS.

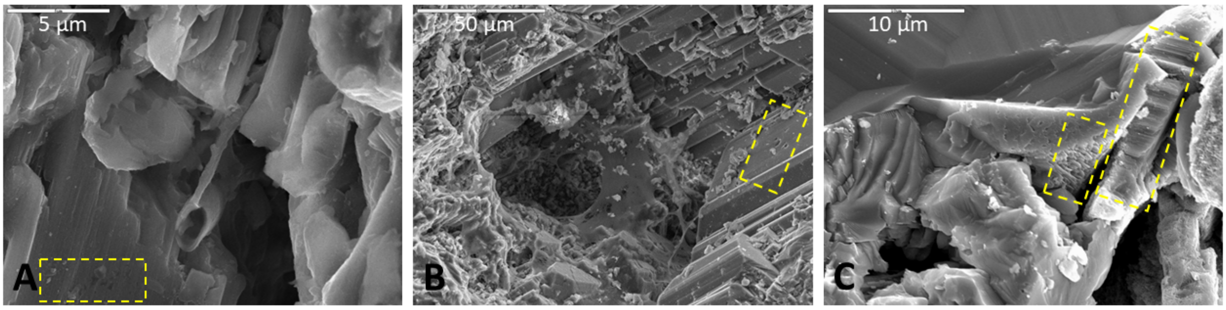

Figure S3. Surface alterations in monzonite showing etch pits and digitate silica structures. Yellow boxes indicate etch pits in panels A and B, and secondary alteration digitate silica structures in C. The images in Panel A is from a chip incubated in Fe carbonate medium, soaked in phosphate buffer, and dried with HMDS; Panel B was incubated in TSI agar, soaked in phosphate buffer and dried by CPD; Panel C was incubated in nutrient broth and air dried.

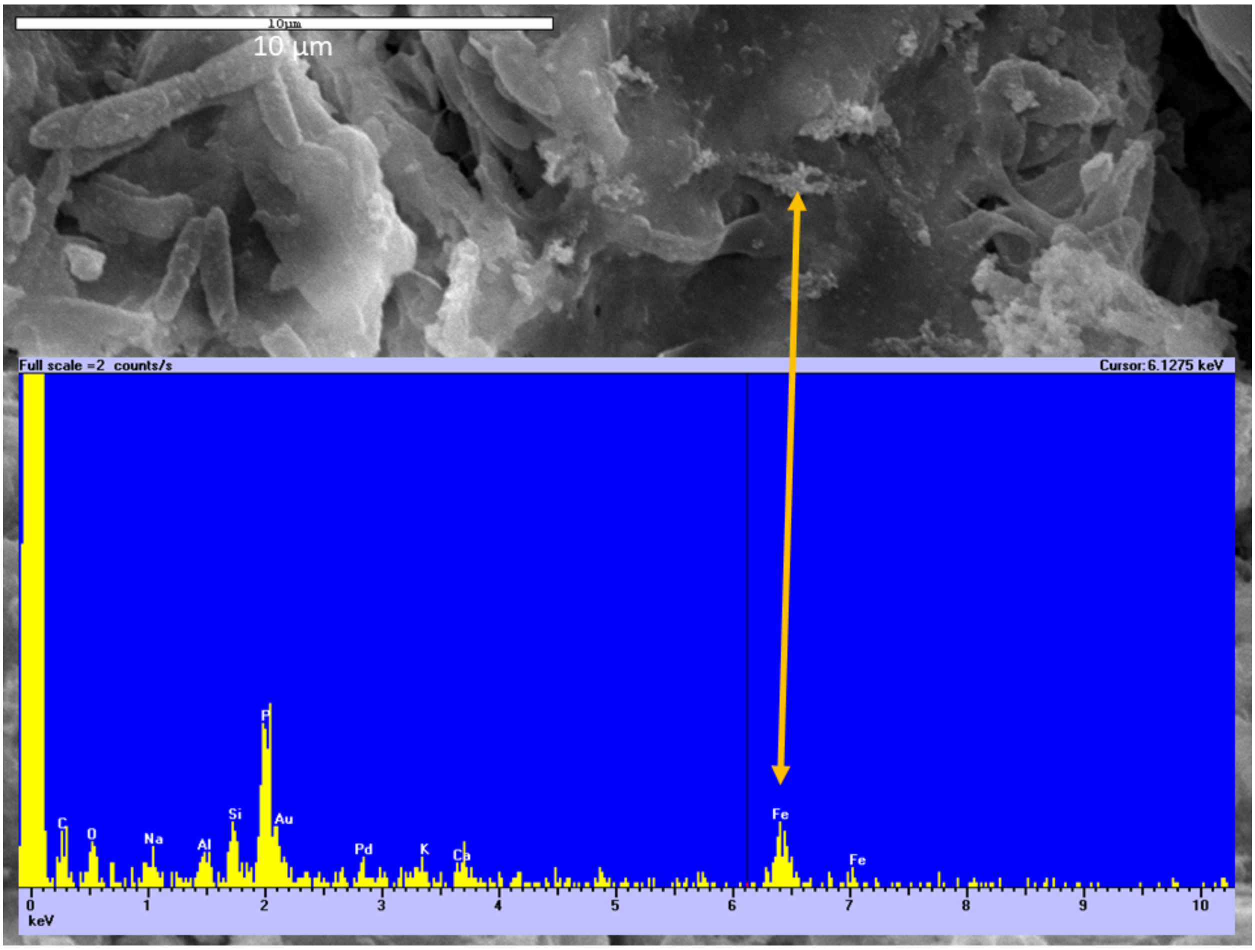

Figure S4. Detail of panel B in Figure 6 in the main text. Yellow arrow in Panel A (top) corresponds to the EDS data for the precipitate incorporated into the biofilm. Phosphorus and iron peaks indicating P–rich and Fe–rich aggregates that have precipitated in the biofilm.

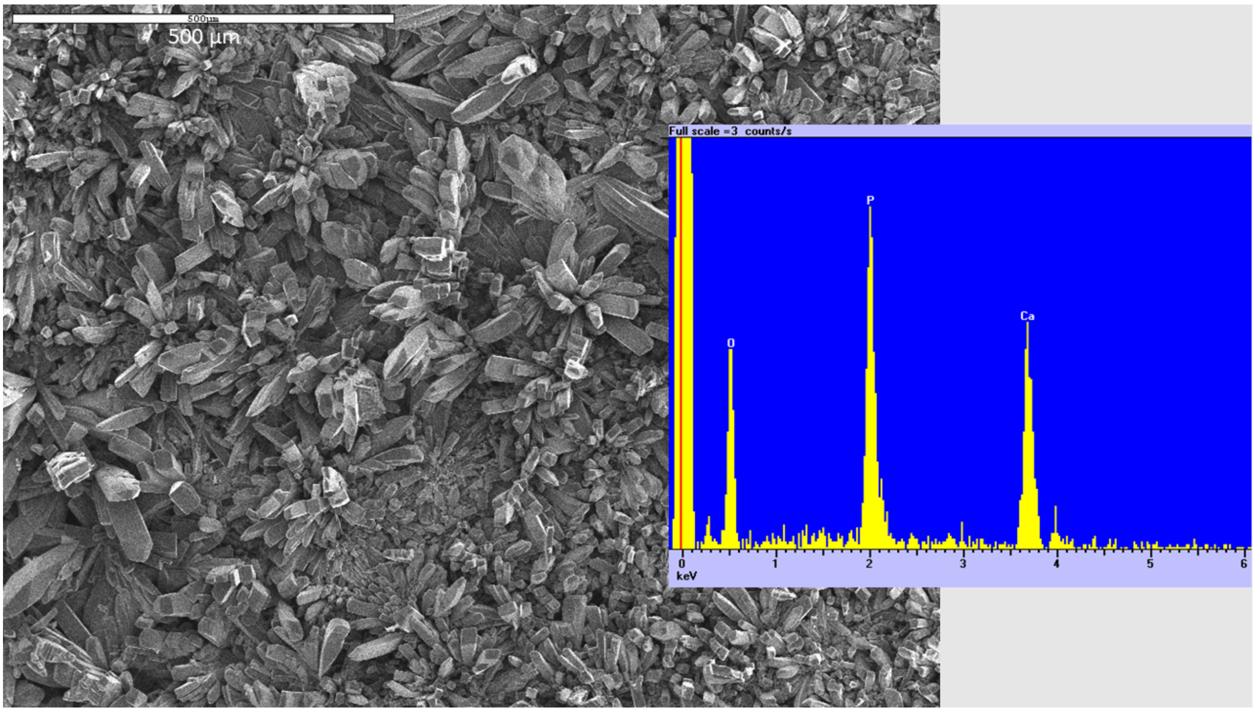

Figure S5. Detail and EDS information associated with panel B from Figure 7 in the main text. Surface of a limestone sample soaked in phosphate buffer shows abundant apatite crystal growth. Scale is 500 µm. Inset shows spectrum on a single apatite crystal, P>Ca>O.

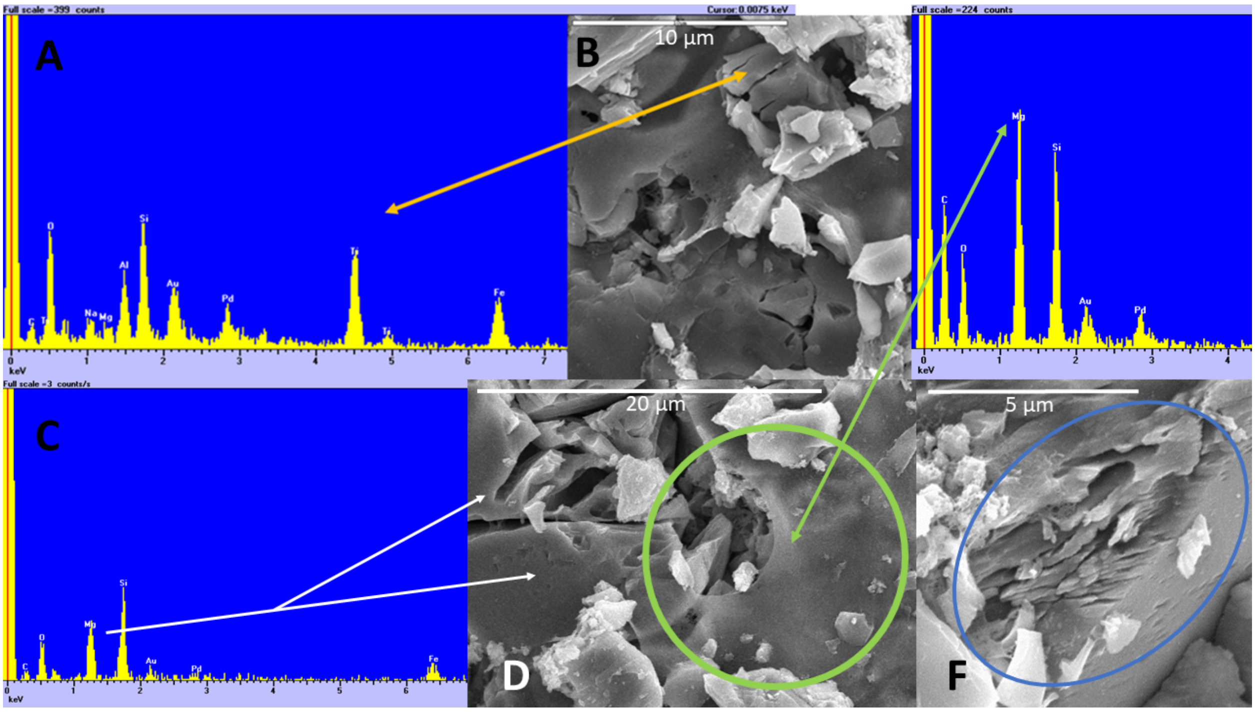

Figure S6. Select panels from the same basalt sample, fixed in glutaraldehyde and dried by HMDS. Panel A: Orange arrow indicates a decrepitated Fe–Ti oxide in Panel B, which shows this basalt sample is highly weathered. The surface in Panel B is also covered with a thick biofilm. Panel D shows dissolution or corrosion of Mg–rich pyroxene (Panel C), indicated by white arrows as referred to in the text. Green circle in Panel D indicates area of a thick, Mg–enriched biofilm (adjacent to the Mg–depleted pyroxene crystals exhibiting dissolution). Note parallel etch pits in pyroxene crystal in Panel D, indicated by the lower white arrow. Panel F shows olivine dissolution along crystallographic planes overlaid by a thick biofilm on top and an adjacent filamentous biofilm (left).

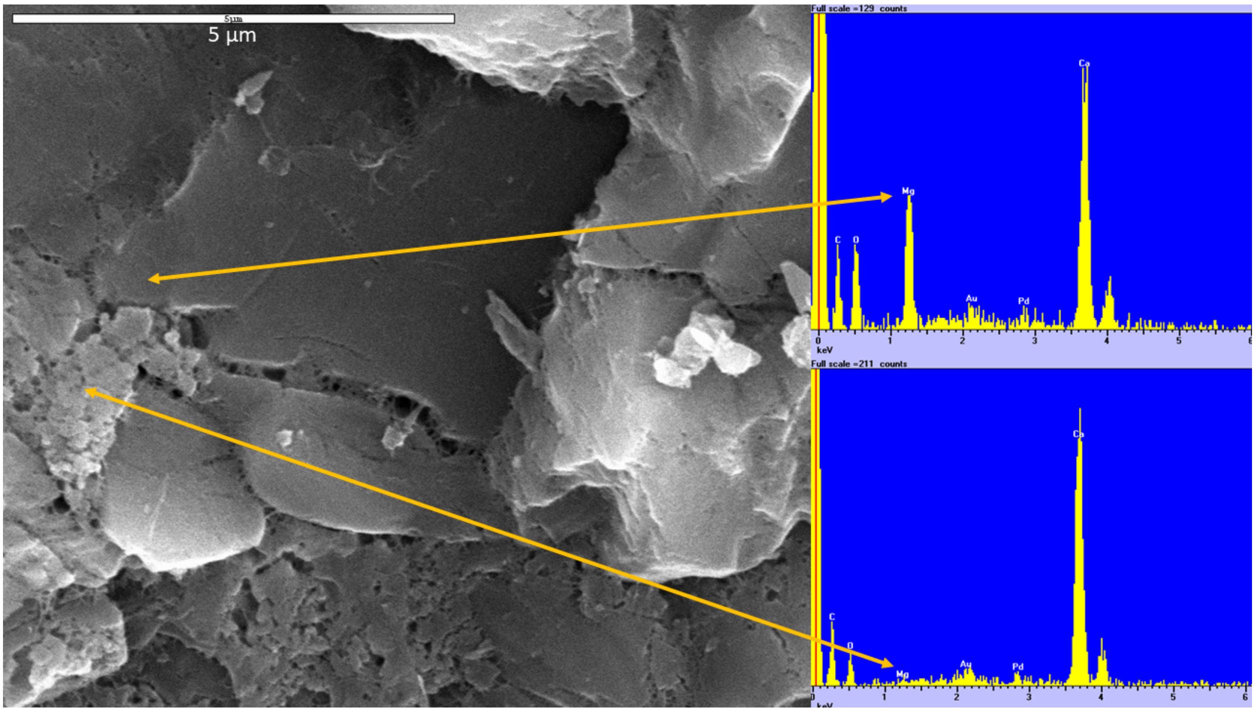

Figure S7. EDS spectra showing Mg depletion in corroded limestone. In what appears to be the same crystal face within a limestone sample, Mg depletion occurs in the corroded portion, but not the uncorroded portion. This limestone billet was incubated in nutrient broth, fixed in glutaraldehyde, and dried via HMDS. Scale bar is 5 µm.

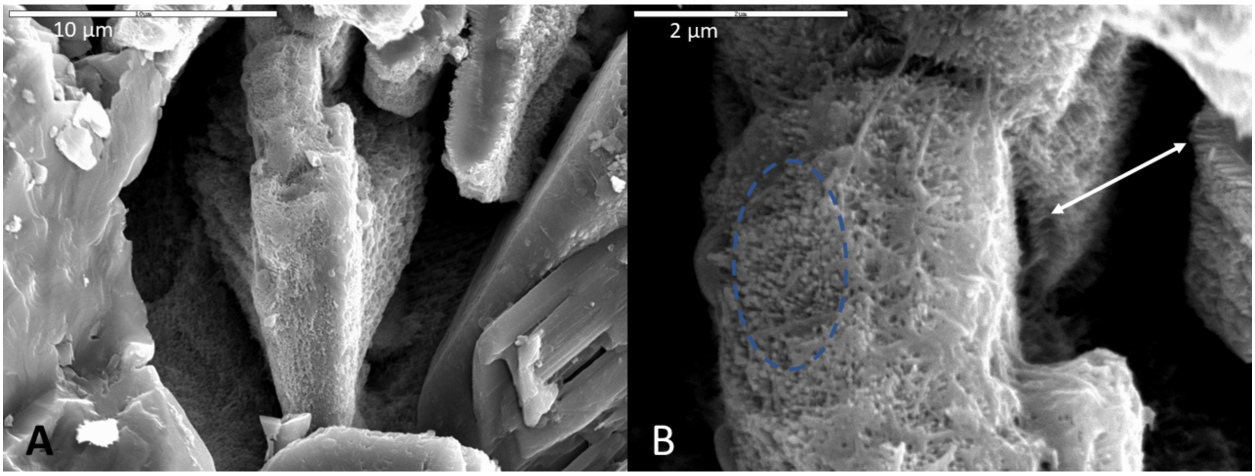

Figure S8. A monzonite sample exhibiting palisades fabric following incubation. The digitate protrusions (inside blue oval) are associated with a filamentous biofilm. The mineralogy of the crystal could not be determined through EDS spectra as biofilm covers the entire surface and EDS returned a high carbon peak. The nearby minerals are quartz (left of palisades fabric–coated mineral in panel A) and corroded feldspar (lower right in A). (A) shows the crystal covered in palisades fabric at 10 µm. (B) shows the same crystal at 2 µm. The white arrow indicates cross–sectional views of the palisades fabric. This monzonite sample was incubated in nutrient broth and air–dried.

Table S1. Saturation indices (SIs) for media used in the long–term incubation experiments

		**Fe carb**	**Mn carb**	**TSI**	**AI**
Phase	***Formula***	***SI***	***SI***	***SI***	***SI***
Artinite	MgCO3:Mg(OH)2:3H2O	–10.9	–11.0		–5.7
Birnessite	MnO2		–12.8		
Bixbyite	Mn2O3		–10.0		
Brucite	Mg(OH)2	–6.7	–6.7		–4.0
Epsomite	MgSO4:7H2O	–4.0	–4.0		–3.7
Fe(OH)2.7Cl0.3	Fe(OH)2.7Cl0.3	5.9	6.0	10.4	11.4
Fe2(SO4)3	Fe2(SO4)3	–40.8	–40.8	–38.0	–37.0
Fe3(OH)8	Fe3(OH)8	–0.4	–0.4	11.0	14.2
Ferrihydrite	Fe(OH)3	0.9	0.9	5.0	6.3
Goethite	FeOOH	5.3	5.3	9.4	10.7
Halite	NaCl			–3.4	–4.1
Hausmannite	Mn3O4		–11.5		
Hematite	Fe2O3	15.6	15.6	23.8	26.3
Hydromagnesite	Mg5(CO3)4(OH)2:4H2O	–26.9	–26.9		–13.7
Jarosite–H	(H3O)Fe3(SO4)2(OH)6	–3.1	–3.1	5.6	8.4
Jarosite–K	KFe3(SO4)2(OH)6	3.7	3.6	11.2	17.1
Jarosite–Na	NaFe3(SO4)2(OH)6				14.4
Lepidocrocite	FeOOH	4.4	4.4	8.5	9.8
Maghemite	Fe2O3	5.2	5.2	13.4	15.9
Magnesite	MgCO3	–3.4	–3.4		–0.8
Magnetite	Fe3O4	16.1	16.0	27.5	30.7
Manganite	MnOOH		–5.0		
Melanterite	FeSO4:7H2O	–5.6	–5.6	–4.1	–4.0
Mg–Ferrite	MgFe2O4	4.9	4.9		18.3
Mirabilite	Na2SO4:10H2O			–3.9	–3.7
Natron	Na2CO3:10H2O			–7.2	–6.4
Mn2(SO4)3	Mn2(SO4)3		–53.7		
Mn3(PO4)2	Mn3(PO4)2		–5.7		
MnCl2:4H2O	MnCl2:4H2O		–11.8		
MnHPO4(C)	MnHPO4		5.8		
MnSO4	MnSO4		–9.3		
Nesquehonite	MgCO3:3H2O	–5.8	–5.8		–3.2
Nsutite	MnO2		–12.2		
Periclase	MgO	–11.4	–11.4		–8.8
Pyrocroite	Mn(OH)2		–5.5		
Pyrolusite	MnO2		–10.5		
Rhodochrosite	MnCO3		–1.5		
Siderite	FeCO3	–2.8	–2.8	0.5	1.1
Strengite	FePO4:2H2O	3.0	3.0		6.2
Thenardite	Na2SO4			–4.8	–4.6
Thermonatrite	Na2CO3:H2O			–8.6	–7.9
Vivianite	Fe3(PO4)2:8H2O	2.2	2.2		9.5
